# Hidden disabilities in patients with autoimmune rheumatic disease: an invisible barrier to daily functioning and healthcare accessibility

**DOI:** 10.1016/j.ero.2025.08.003

**Published:** 2025-09-06

**Authors:** Faisal Parlindungan, Rudy Hidayat, Sumariyono Sumariyono, Suryo Anggoro Kusumo Wibowo, Anna Ariane, Johanda Damanik, Abirianty Priandani Araminta, Cindy Oey, Mitra Alparisa, Indra Saputra, Annisa Aulia Fitri, Rio Rialdi, Tamariska Evelyn

**Affiliations:** 1 Rheumatology Division, Department of Internal Medicine, Faculty of Medicine, Universitas Indonesia, Ciptomangunkusumo National General Hospital, Jakarta, Indonesia; 2 Medical Staff Group of Internal Medicine, Universitas Indonesia Hospital, Depok, Indonesia; 3 Jakarta Rheumatic and Autoimmune Disease Study Group (Jak-RAIDS), Jakarta, Indonesia; 4 Junior Doctor Network-Indonesian Medical Association, Jakarta, Indonesia

## Abstract

**Objectives:**

Patients with autoimmune rheumatic disease (ARD) frequently experience invisible yet disabling symptoms, including chronic pain, fatigue, mobility issues, sensory impairments, and cognitive dysfunction. These hidden disabilities can hinder daily functioning, particularly in accessing healthcare via public transportation. This study explored real-life experiences of patients with ARD, focusing on how their condition affects daily life and perceptions of public transportation accessibility for hospital visits in Jakarta.

**Methods:**

A qualitative study using focus group discussions was conducted in May 2024 with 14 patients with ARD aged 18 to 60 years who regularly used public transportation in Jakarta. All participants had nonvisible disabilities. Individuals using mobility aids, holding disability identifiers, or pregnant were excluded.

**Results:**

The primary theme focused on participants’ perceptions of their disease and treatment, highlighting the impact on daily life, unpleasant hospital visit experiences, and coping attitudes. The second theme focused on accessibility of public transportation, emphasising issues with difficulties in using public transportation, infrastructure, and personnel awareness. Third, participants discussed the importance of identifiers for hidden disabilities, including methods for identification, disability groups categorisation, and attitudes towards advocacy efforts for hidden disability.

**Conclusions:**

Hidden disabilities profoundly affect patients’ with ARD daily lives, particularly their ability to navigate public transportation. Patients with ARD were not regarded as a priority group in public transportation, which hindered their access to healthcare facilities. Introducing identifiers for individuals with hidden disabilities can enhance public awareness and improve accessibility, supporting better traveling experiences in public transportation for this group.

## INTRODUCTION

Many individuals with disabilities live with invisible conditions that lack visible symptoms or signs, including those with autoimmune rheumatic diseases (ARDs). ARD is a group of chronic illnesses that can significantly impact the quality of life of those affected. This group includes systemic lupus erythematosus (SLE), rheumatoid arthritis (RA), Sjogren disease (SjD), systemic sclerosis (SSc), spondyloarthritis (SpA), and other connective tissue diseases [1]. Individuals with ARD face disabilities that are not immediately apparent to an observer, often referred to as hidden disabilities, which can pose challenges in their daily lives.

Individuals with hidden disabilities may appear visually healthy and functioning in their daily lives without any visible aids, despite experiencing a range of symptoms such as sensory impairment, chronic pain, cognitive dysfunction, or mental health conditions. Healthy individuals in their surroundings might perceive the joint pain and fatigue experienced by patients with ARD as a typical postwork tiredness, causing them to endure severe discomfort without others recognising the true extent of their suffering. Several studies have reported the subjective experiences of individuals with ARDs, highlighting their impact on daily life. These include reduced physical capacity and well-being, psychological and emotional challenges, cognitive dysfunction, and difficulties in fulfilling normal social roles. The unpredictable nature of ARD symptoms further contributes to disruptions across nearly every aspect of daily life [2,3].

In recent decades, the prevalence of ARD has increased globally, including in Indonesia. ARD is estimated to affect around 5% of the general population [4]. SLE is one of the most common ARDs in Indonesia, and data from the Indonesian Rheumatology Association showed that the proportion of patients with SLE visits to rheumatology clinics in hospitals across Indonesia increased from 17.9% to 27.2% in 2015 to 30.3% to 35.8% in 2017. These figures represent the percentage of SLE visits out of rheumatology clinic visits nationwide [5]. The prevalence of ARD in Indonesia has risen due to improved diagnostic capabilities and enhanced patient reporting and record-keeping, as well as environmental factors such as COVID-19 infection.

Jakarta is a city known for its heavy traffic and diverse modes of transport, including the bus rapid transit system (TransJakarta), electric rail trains (Commuter Line), the Jakarta Mass Rapid Transit and Light Rapid Transit, and local minibuses (JakLingko). As a metropolitan city, Jakarta continues to evolve to provide integrated and accessible public transportation for its citizens. A survey by Greenpeace Indonesia found that public transportation is the most popular mode of travel, accounting for up to 40% of total respondents [6].

For individuals with ARD, safe and comfortable public transport is crucial to ensure adherence to medical treatment. The accessibility of public transport can also be a determining factor in the success of the therapy. A study in Jakarta identified transportation challenges as a major factor contributing to the discontinuation of follow-up hospital visits among patients with RA [7].

Healthcare provision in Jakarta primarily relies on centre-based services, particularly for patients with ARD, as Indonesian rheumatologists are currently available only in tertiary hospitals located in major cities with well-developed public transportation systems, such as Jakarta. Access to ARD-specific medications and advanced laboratory or radiologic examinations is also limited to these urban centres, making transportation accessibility a crucial factor in patient adherence to hospital visits.

The healthcare system of Indonesia operates largely under the Badan Penyelenggara Jaminan Sosial Kesehatan (BPJS), the national health insurance system for Indonesians, providing universal health coverage for the majority of its citizens. The system follows a tiered referral mechanism, in which patients must first visit primary healthcare facilities to obtain referrals to secondary or tertiary hospitals for specialist care, including rheumatology. As of now, telemedicine is not yet well-established in Indonesia, leaving in-person hospital visits as the primary option for receiving specialist care; therefore, accessibility to public transportation is very important for the patients.

This study addresses the urgent need to understand the experiences of individuals with ARD in accessing public transportation in Jakarta. Up until now, no initiatives in Indonesia have allowed hidden disability groups to voice their opinions. This study serves as a tool to advocate for the rights and needs of individuals with hidden disabilities, especially those diagnosed with ARD, to the relevant stakeholders. Investigating their perceptions of hidden disabilities can provide valuable insights for developing inclusive policies and solutions. Enhancing transportation accessibility may ultimately improve mobility and healthcare access for individuals with ARD.

## METHODS

**Figure 1:**
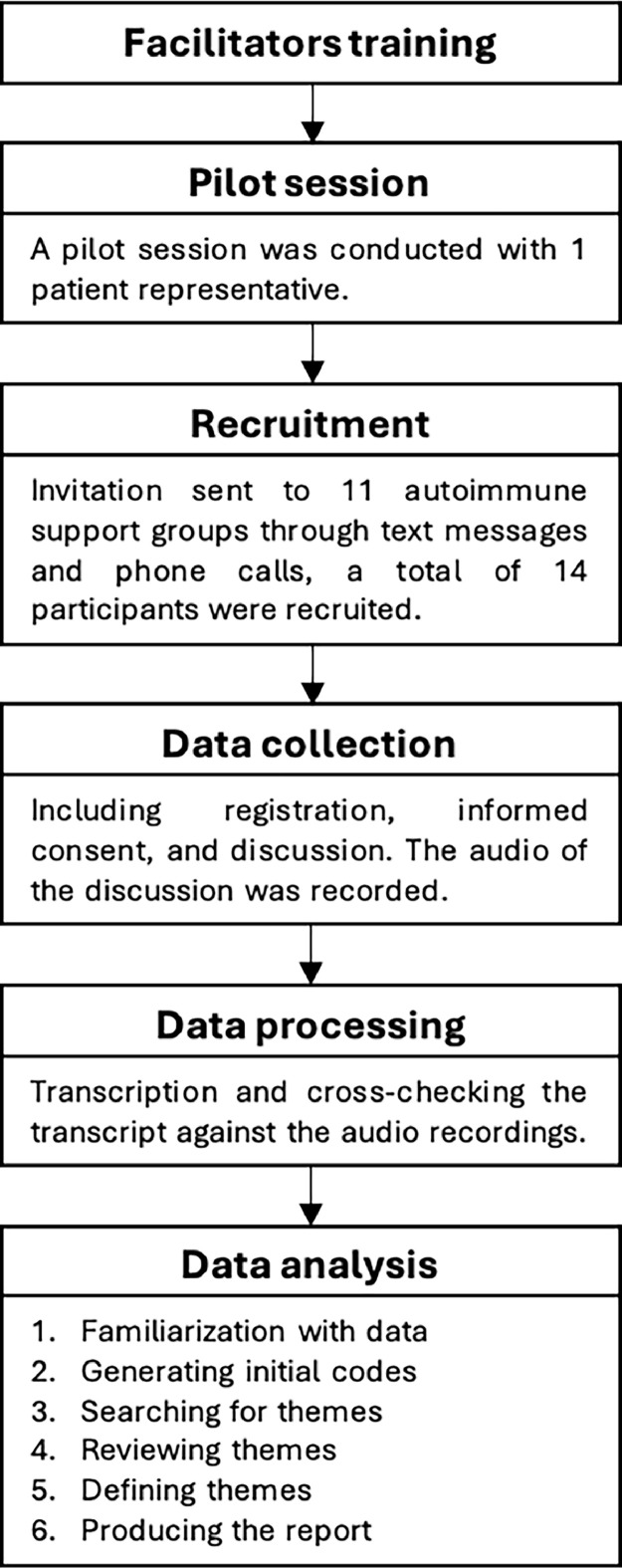
Study flowchart.

The aim of this study was to explore the real-life experiences of individuals with ARD, focusing on how their condition affects daily life and their perceptions of the accessibility of public transportation for hospital visits in Jakarta. Included were patients diagnosed with ARD, aged between 18 and 60 years, who frequently used public transportation for hospital visits in Jakarta. Exclusion criteria included individuals who used mobility aids, were pregnant, or had previously received disability identifiers due to visible disabilities such as visual impairment, deafness, paralysis, and other similar conditions. These identifiers could take the form of pins, badges, or priority access cards. The study flowchart is shown in Figure 1.

### Pilot session

A focus group discussion (FGD) was conducted to collect data. The discussion was facilitated by 3 doctors, who included a chief facilitator and 2 supporting facilitators. All facilitators underwent training on how to conduct FGDs and subsequently developed a set of pilot questions for patients. The set was also discussed with 1 patient with ARD to ensure the clarity of the questions.

The group size was carefully managed using structured discussion facilitation techniques to ensure equal participation, including the following:

1. Directed questioning to involve quieter participants
2. Rotation-based responses to ensure engagement
3. Breakout reflections before open discussions
4. The focus group guide was developed based on preliminary consultations with autoimmune patient support groups.
5. A pilot session with one patient representative lead to minor revisions in question phrasing to improve clarity and inclusivity.

### Recruitment

Invitations for the FGD were sent to 11 autoimmune support groups through text messages and telephone calls. The support groups were requested to send their representatives to attend the FGD. Preparation included arranging necessary equipment such as audio recorders, seating plans, registration forms, and writing materials. The invitation was also extended to photographers to help document the event.

Eight groups responded to the invitations, including Syamsi Dhuha Foundation, Yayasan Skleroderma Indonesia, Sahabat Rheumatoid Arthritis, Komunitas Autoimun Indonesia, Komunitas Lupus Sehati, Relawan Lupus Indonesia, Yayasan Lupus Indonesia, and Yayasan Marisza Cardoba Foundation. These were selected due to their widespread membership across Indonesia, representing individuals with various autoimmune diseases. They are also actively involved in advocacy efforts and initiatives related to autoimmune disease awareness. A total of 14 participants were recruited using purposive sampling.

### Data collection

The FGD commenced with a registration process lasting for an hour, during which participants provided written consent. Following registration, a briefing was conducted, including study background, objectives, and discussion guidelines. Participants were assigned to their respective seats in a meeting room to ensure adequate space and comfort. The FGD transpired on a single day from 3:00 PM to 5:00 PM.

Audio recorders were strategically positioned to capture discussions clearly, and periodic checks ensured uninterrupted recording. Facilitators adhered to the discussion timeline, ensuring that all questions were covered. Each participant was allowed to share additional thoughts before the session concluded. The FGD lasted for 2 hours, and key findings were summarised.

### Data processing

Immediately after the FGD, the notetaker transcribed the discussions at the event venue. Observations, immediate reflections, and any clarifications on participants’ responses were noted. The primary researcher then cross-checked the transcriptions against the audio recordings to ensure data accuracy.

### Data analysis

The collected qualitative data were analysed using both inductive and deductive approaches. Thematic analysis was performed using NVivo 14 software, following the 6-step framework of Braun and Clarke (2006):

1. Familiarisation with data: transcripts were reviewed, and key points were noted.
2. Generating initial codes: recurrent patterns were coded systematically.
3. Searching for themes: codes were grouped into categories and broader themes.
4. Reviewing themes: relationships between themes were mapped.
5. Defining themes: subthemes were refined, ensuring alignment with study objectives.
6. Producing the report: themes were structured into narrative findings. We used the Consolidated Criteria for Reporting Qualitative Research Checklist to verify the items included in the qualitative research report [8].

The FGDs were conducted and transcribed in Bahasa Indonesia. Two coders ensured accuracy in generating the codes. The analysis was performed using the original transcripts, and illustrative quotes presented in the manuscript were translated into English. This analysis helped identify frequently mentioned perceptions about accessibility to public transportation among patients with ARD in Jakarta.

### Study approval

The Ethics Committee of the Faculty of Medicine, Universitas Indonesia—Cipto Mangunkusumo Hospital reviewed and approved this study on April 5, 2024, with protocol number 24-03-0467.

## RESULTS

### Study participants

This FGD involved 14 participants who are patients with a variety of ARD. The profile of the research participants is shown in Table 1. One participant (P7) was excluded from the FGD because she presented with a visible disability (low vision with a walking aid).

**Table 1:** Sociodemographic profile of focus group discussion participants

Key informants	Gender	Age (y)	Autoimmune rheumatic disease
P1	Female	58	SLE
P2	Male	45	SLE
P3	Female	36	SpA, SjD
P4	Female	50	SLE
P5	Female	56	RA
P6	Female	44	SSc, ILD
P7	Female	59	SLE with low vision
P8	Female	54	SpA
P9	Female	37	SSc
P10	Female	37	SLE
P11	Female	51	SLE
P12	Female	46	RA
P13	Female	44	SLE
P14	Female	44	SLE

### Themes

This FGD identified 3 themes that related to the following: (i) patients’ with ARD perceptions of their disease and treatment, (ii) patients’ with ARD views on current public transportation accessibility in Jakarta, and (iii) the use of special identifiers for recognising hidden disability groups in public transportation. Notable quotations from study participants for each theme and category are shown in Table 2, Table 3, Table 4.

**Table 2:** Categories and participant quotes of the first main theme: subjects’ perceptions of their disease and treatment

Category	Main idea	Interpretation	Participant quote
Impact on daily life	Changes in daily routine	Pain and the anticipation of falling could slow down participants’ pace in daily activities	‘Since I was diagnosed with autoimmune disorders, my activities have slowed down because I need to be careful not to fall and to reduce excessive pain when doing tasks at the usual speed.’ [Interviewee P1, Female, 58, SLE]
		Additional routines would slow down daily pace	‘What I feel is that because of the treatments needed, especially like preparing more suitable food to support our disease management, my daily routine has changed. … and that automatically takes up part of my time. If we compare it with healthy friends who don't need medication or anything like that, my speed slows down because of those additional activities.’ [Interviewee P2, Male, 45, SLE]
		Slower cognitive function had negative effect on daily work performance	‘For me, besides physical activity, my cognitive ability has decreased. So, if before I could easily understand something, after developing an autoimmune condition, I need to repeat things two or three times to understand simple concepts. This has affected my school and work performance.’ [Interviewee P3, Female, 36, SpA, SjD]
	Physical burden	Difficulties in using stairs at train stations	‘When I want to go downstairs, it feels like a struggle, and as I walk slowly, people behind me push or rush me, even though I'm trying hard to get down... and I also have to focus on each step while watching the stairs carefully as I move my feet.’ [Interviewee P8, Female, 54, SpA]
		Demonstrating their ‘disabilities’ to gain access to priority seating	‘When I'm not feeling well, I carry around my large CT scan results so that people know that I’m a patient and they give me their seats. Otherwise, I wouldn't be able to get one.’ [Interviewee P9, Female, 37, SSc] ‘With the condition I feel right now and using knee braces every day, I often have to lift my pants to show the braces so that people can see it better.’ [Interviewee P5, Female, 56, RA]
		Physical condition as a determinant of travel speed	‘It depends on my condition. If I'm feeling fit, it can take me 45 minutes to an hour if there's no traffic. If there's traffic, it can take up to 1.5 hours. … When I drive my motorcycle, my hands often become stiff due to SSc, and I experience numbness, so I have to pull over for a while.’ [Interviewee P9, Female, 37, SSc]
	Emotional burden	Psychological challenges in everyday life	‘Since being diagnosed with an autoimmune condition, I have been more emotional and sensitive than before.’ [Interviewee P4, Female, 50, SLE] ‘… Regarding our mental health, particularly from what I've observed, many people share their experiences about being left by their partners. This highlights that we truly have a hidden disability, so we need to find ways to manage our mental health at home, as it significantly impacts our daily activities and treatment.’ [Interviewee P6, Female, 44, SSc, ILD]
		Coping with negative opinions regarding health conditions as a part of managing mental health	‘I often become the centre of attention due to the changes in my face, especially before the pandemic, when it wasn’t common for people to wear masks in public. Sometimes, there is a feeling of shame and a lack of confidence that affects my mental health, which makes me avoid seeking treatment. … Maybe other people doesn’t mean to judge me, but they may have thought, “Oh, she looks so unique.” That’s how I choose to perceive it, by staying positive.’ [Interviewee P6, Female, 44, SSc, ILD]
		Impacted ego as a male with ARD	‘So when I have to regularly consult the doctor, people see me as healthy, with no apparent issues. But in front of my wife and kids, it's like, “Why are you always going to the doctor?” That's where my ego as a man gets tested... I prevent myself from getting sick. Because if I do, it'll worsen to the complaint, like “You go to the doctor so often, but you're still sick.” What I feel is a bit different, given my position as a man.’ [Interviewee P2, Male, 45, SLE]
Unpleasant hospital visit experiences	Issues with long queues	Long queues are exhausting	‘I've been a patient in this hospital for 3 months, and every time I want to pick up my medication after the doctor consultation, the wait in the pharmacy is incredibly long... it takes nearly 6 hours before I can get my medication.’ [Interviewee P11, Female, 51, SLE] ‘By the time I get home from the hospital, I'm completely exhausted. It is so stressful and I feel traumatized every time I need to go pick up my medication.’ [Interviewee P11, Female, 51, SLE]
		Discomfort in queueing while the disease is flaring up	‘What often happens is that I frequently end up sitting on the hospital floor while waiting in line. If I'm feeling okay, I can accept it, but when I'm in pain or not feeling well, it makes it really hard for me to stand up, and I end up stiff and struggling to walk.’ [Interviewee P3, Female, 36, SpA, SjD]
		The use of Badan Penyelenggara Jaminan Sosial Kesehatan (BPJS) or the National Health Insurance resulting in longer queues	‘If I have to use BPJS, then I have to spend a whole day.’ [Interviewee P1, Female, 58, SLE] ‘My experience waiting in line at the hospital as a BPJS patient is not pleasant because the queue is so long.’ [Interviewee P3, Female, 36, SpA, SjD]
		Telemedicine as an option of seeking treatment	‘So, the obligation to do follow-up doctor appointments really depends on what stage we are in. If I’m at a stage where I need to see the doctor, I will prioritize the treatment. But if I’m feeling fit, I tend to prioritize other things over the follow-up appointments and start utilizing the available facilities. After the pandemic, treatment options have started to open up, such as telemedicine.’ [Interviewee P1, Female, 58, SLE]
Coping attitudes	Lifestyle changes	Adopting healthy habits	‘I have made an effort to eat with a healthy lifestyle, … and because of that, I have tried to avoid the foods that the doctor said should be avoided.’ [Interviewee P5, Female, 56, RA] ‘In the morning, I exercise first, then prepare healthy meals to take with me …’ [Interviewee P2, Male, 45, SLE]
		Keeping the motivation to seek treatment	‘I feel that going to the doctor is obligatory for me, because I believe that being given this kind of illness means I am being given a chance by God to fight... Whatever I do, I want to heal. … I need to invest in my long-term health. After all, who will love ourselves if not us?’ [Interviewee P8, Female, 54, SpA] ‘Even though my laboratory results showed improvements and the pain in my fingers subsided, but often times the pain come and go. The pain could be extremely painful. That’s why medication is a priority for me to control the disease activity, and no matter what, I have to make it to the hospital.’ [Interviewee P5, Female, 56, RA]

**Table 3:** Categories and participant quotes of the second main theme: subjects’ perceptions of current public transportation accessibility

Category	Interpretation	Participant quote
Difficulties in using public transportation	Physical condition as a consideration for the mode of transportation	‘I feel that using public transportation is very uncomfortable. I often avoid public transport during rush hours.’ [Interviewee P10, Female, 37, SLE] ‘My mode of travel depends on my condition; if I’m feeling fit, I can use public transportation, but if I’m not feeling well, I use online transportation services. Although online transportation services isn't entirely comfortable either, …. On the other hand, using online transportation services is higher in costs and the traffic jams also mentally exhaust me.’ [Interviewee P3, Female, 36, SpA, SjD] ‘I usually travel by motorcycle taxi because if I don't use one, I won't make it (to the doctor) on time. I no longer use the train (Commuter Line) because of issues with my legs, so I don't feel confident using it unless I have a friend with me.’ [Interviewee P4, Female, 50, SLE]
	Distance between TransJakarta bus stops is too far apart	‘When taking the busway, considering my residence is near the border between Depok and Jakarta,… the number of bus stops should be added.’ [Interviewee P6, Female, 44, SSc, ILD]
	Overcrowding of Commuter Line trains and limited train routes	‘… the expansion of railway lines is still limited. The lines don't connect widely or reach the more remote areas.’ [Interviewee P2, Male, 45, SLE] ‘The Commuter Line train … is always crowded.’ [Interviewee P9, Female, 37, SSc] ‘… is a big struggle because of the long lines and overcrowding. The number of train units needs to be increased.’ [Interviewee P2, Male, 45, SLE]
Public transportation infrastructure	Lack of clear directional signs	‘For example, at Depok Baru station, it seems like only people who access the station daily understand the area well. When someone goes there for the first time, they can easily get confused and will need to ask for directions.’ [Interviewee P2, Male, 45, SLE]
	Visual aids should be clearly visible	‘If there is a shortage of human resources, a solution proven effective in other countries providing clear signage. If the signs are truly clear and comprehensive, even if it's your first time visiting the area and you don't ask anyone, you can easily know where to go and exactly move to the designated points.’ [Interviewee P2, Male, 45, SLE] ‘In terms of signage, such as indicators for turning right, restrooms, and other directions, the current signs are perceived as unhelpful due to the choice of color combinations, like blue and white, which are not easily visible from a distance. It would be better to use black and white instead of blue and white, as those colors are not sufficiently contrasting.’ [Interviewee P1, Female, 58, SLE]
	Escalators are often found out of service	‘While commuting at the Commuter Line train stations like Manggarai or Bekasi, the escalators are often found to be turned off, whether for going up or down. So I have to struggle to climb up the stairs, as the escalators remain out of service.’ [Interviewee P5, Female, 56, RA]
	Building designs do not meet the specifications they should	‘At the TransJakarta bus stops, the quality and type of the used flooring materials do not meet the ideal specifications for outdoor use. If rainwater splashes onto the floor, it poses a risk of fall. The tiles used on the building are not meant for outdoor use, cracked, and installed in an improper pattern, potentially leading to water pooling.’ [Interviewee P2, Male, 45, SLE] ‘Sometimes the distance between the bus and the bus stop is quite far.’ [Interviewee P1, Female, 58, SLE] ‘… the floor is slippery and there are no handrails … At Depok Baru station, there are steps that are quite high and uneven, and there are stairs without handrails.’ [Interviewee P8, Female, 58, SpA] ‘The varying heights of the stairs often pose challenges, especially for those with knee and hip issues. When the distance between the steps is too short or the width of the stairs is not appropriate, it becomes difficult to place one’s foot securely.’ [Interviewee P1, Female, 58, SLE]
	The need for standardised building designs in every region to ensure functionality and accessibility	‘Another finding is regarding design standardization. Each region often creates its own designs. While this is not a problem and shows creativity, there still needs to be a standardization used. For example, seating made of stainless steel is very slippery. The variation in design with three bars spaced apart makes it difficult for people to sit.’ [Interviewee P2, Male, 45, SLE] ‘Additionally, from a visual standpoint, in situations where there are visual impairments (such as myopia or hyperopia), visuals that appear Instagram-worthy may not necessarily be functional or easily visible.’ [Interviewee P1, Female, 58, SLE]
Public transportation personnel awareness	Shortage of personnels and the staffs are not proactive in assisting passengers with special needs	‘So far, the staff tends to wait to be asked for help rather than being proactive in monitoring which passengers might need assistance and identifying potential risks.’ [Interviewee P2, Male, 45, SLE] ‘It's not just the architectural and infrastructural aspects that need attention. A key point to note is the human resources as the main power—staff members who are attentive and proactive in observing and identifying individuals with hidden disabilities or those experiencing physical changes.’ [Interviewee P1, Female, 58, SLE]

**Table 4:** Categories and participant quotes of the third main theme: the use of identifiers for hidden disability groups

Category	Interpretation	Participant quote
Perceptions toward disability groups categorisation	Insights regarding other priority groups	‘From my experience on the Commuter Line, pregnant women can just say "Pregnant woman, pregnant woman!" and immediately get a seat. Some people who was pretending to be asleep would stand up because the security will help find them a seat. I feel a bit envious. Am I supposed to carry a pregnant identification card? That's just not possible.’ [Interviewee P5, Female, 56, RA] ‘When using public transportation, if someone doesn't appear sick from the outside, it will be difficult to get a priority seat.’ [Interviewee P10, Female, 37, SLE]
	General public's lack of understanding of the hidden disability group	‘In public transportation and during daily activities, I often feel out of breath easily, but others are unaware that I am feeling this because of the fatigue from standing on the train or bus.’ [Interviewee P6, Female, 44, SSc, ILD] ‘However, since I appear healthy and fit, I always end up standing and can't get a seat in public transportations.’ [Interviewee P9, Female, 37, SSc] ‘If I mention that I'm feeling unwell to ask for a seat, I'm afraid it will cause a scene, and someone might record it and make it go viral, so I end up saying nothing and just giving up the seat.’ [Interviewee P10, Female, 37, SLE]
Methods for identification	Concerns regarding the potential misuse of the special identifiers	‘We do need and require special identifiers for hidden disabilities, but on the other hand, those identifiers could be misused by other people who do not have hidden disabilities.’ [Interviewee P3, Female, 36, SpA, SjD] ‘There are still people who misuse disabilities. If we are given special identifiers, we might become easy targets. Friends who use wheelchairs have shared experiences of being victims of irresponsible individuals, whether it be having their belongings taken, facing aggressive behavior, or even sexual harassment.’ [Interviewee P1, Female, 58, SLE]
	Special identifiers should be safe and secure to prevent misuse	‘If the system is integrated with the Ministry of Health, and we are identified through our organizations, it would be more secure. Members of these organizations could be registered with Ministry of Health, and we could be recognized in their database, ensuring greater safety.’ [Interviewee P1, Female, 58, SLE] ‘For identification purposes, it could be designed similar to a JakLingko card or registered with unique data. A proper system could be established to ensure it cannot be duplicated. It might use ID cards (KTP) or a special transportation card that has already been registered with the Ministry of Health.’ [Interviewee P3, Female, 36, SpA, SjD] ‘I personally prefer using a card without any visible identification. We could carry an identifier card to show when sitting in priority seats … Sometimes, people who are not part of priority groups sit there. We could simply show the card to indicate that we have the right to sit there, because it has the logo. Once we put the card away, others won’t see us as someone with autoimmune disease or a disability anymore. This conventional approach might be easier to implement, safer, and still effective.’ [Interviewee P2, Male, 45, SLE] ‘Patients who genuinely need the card must be properly registered, to prevent any misuse.’ [Interviewee P4, Female, 50, SLE]
Subjects' attitudes toward the hidden disability advocacy campaign	Enthusiasm and high hopes of participants	‘…, when I heard about this FGD, I was very excited to attend it.’ [Interviewee P2, Male, 45, SLE] ‘With this FGD, it is hoped that there will be encouragement and pressure to the official institutions, leading to significant changes.’ [Interviewee P2, Male, 45, SLE]

### Theme 1: Participants’ perceptions of their disease and treatment

#### Category 1: Impact on daily life

##### Changes in daily routine

The participants acknowledged that having ARD impacted their daily tasks and work. They experienced slower cognitive processing and a reduced pace in completing everyday activities. Participants shared that pain and the anticipation of falling, along with additional routines that require extra time, would slow down their overall pace. Slower cognitive function also negatively affected participants’ daily work performance.

##### Physical burden

Commuting is an important daily activity; however, individuals with ARD face significant challenges due to physical limitations. Participants struggled with stairs at train stations due to chronic pain, which could be disabling yet invisible to others. When using public transportation, they often found themselves needing to prove their ‘disabilities’ to access priority seating.

These physical limitations also significantly impacted private transportation, as one respondent explained that her condition (SSc) affects the time taken for covering a certain distance, making it a key determinant of her travel speed.

##### Emotional burden

Beyond physical limitations, patients with ARD also experience emotional and psychological changes. Patients with SSc often experience visible physical changes on their faces and limbs, along with hidden symptoms such as shortness of breath from interstitial lung disease (ILD). One respondent described the mental burden of coping with negative opinions about her condition, whereas a male participant, as a family breadwinner, expressed concerns that frequent doctor visits could affect his sense of pride within his family.

#### Category 2: Unpleasant hospital visit experiences

Hospital visits are often exhausting for patients with ARD due to long queues for doctor appointments and medication collection. Fatigue and chronic pain, especially during flare-ups, make rest essential after each visit. BPJS is a government agency responsible for universal healthcare for Indonesians. It covers a vast number of participants, further contributing to wait times. Given the challenges faced during each hospital visit, patients experience a dilemma regarding their obligation to seek treatment. When in remission, seeking care may become less of a priority, leading a participant to opt for telemedicine as an alternative to in-person visits.

#### Category 3: Coping attitudes

Lifestyle changes are also essential for patients with ARD to optimise their treatment outcomes, including adopting healthy diets and exercising regularly. Living with ARD presented a significant challenge for patients. The participants agreed that they must prioritise their health as a long-term investment. Seeking healthcare providers is essential, so they have to stay motivated to continue receiving treatment.

### Theme 2: Participants’ perceptions of current public transportation accessibility

#### Category 1: Difficulties in using public transportation

Traveling by public transport is challenging for patients with ARD, especially during peak hours. The mode of transportation chosen to reach the hospital varies depending on their physical condition and other health reasons.

On the basis of the experiences of the respondents, the distance between TransJakarta bus stops in several areas was considered too far apart. Many rely on the Commuter Line, but overcrowding and limited route coverage create difficulties, emphasising the need for additional trains and expanded routes.

#### Category 2: Public transportation infrastructure

The design of bus stops and train stations is crucial in enhancing accessibility for public transport users. A common issue faced was the lack of clear directional signs indicating the platform or gate to use and the location of escalators or elevators. Some respondents suggested a solution to address this issue by maximising the use of visual aids. Visual signs should be clearly visible, with designs readable from a distance.

Escalators are helpful aids that support the mobility of patients with ARD with joint pain, reducing the burden on their joints. However, at several train stations, escalators often did not function, forcing patients to use the stairs while dealing with pain.

The design of bus stops and stations was intended for outdoor use; however, they often fail to meet proper accessibility standards. Several respondents highlighted issues such as large gaps between platforms and buses, uneven step heights, and slippery floors without handrails. These flaws significantly challenged patients with joint pain or mobility issues, increasing their risk of falls.

#### Category 3: Public transportation personnel awareness

According to the respondents, current problems regarding public transport staff include a shortage of personnel and a perception that the staff are not proactive in assisting passengers who need help. The staff did not understand the existence of a hidden disability population, resulting in delayed or inadequate assistance.

### Theme 3: The use of identifiers for hidden disability groups

#### Category 1: Perceptions toward disability group categorisation

Patients with ARD, as individuals with hidden disabilities, often feel overlooked as a priority group in public transportation. The respondents also shared their insights regarding other priority groups, such as pregnant women and the elderly. Despite having hidden disabilities, patients with ARD were often perceived as healthy. This misconception arises from others’ lack of understanding of their struggles with discomfort and pain, which may not be apparent to those around them.

#### Category 2: Methods for identification

Some respondents expressed concerns about providing special identifiers for hidden disability groups on public transport. They worry that such identifiers could be misused by those who falsely claim to have a hidden disability to gain priority access. While special identifiers might help, they could also increase the vulnerability to targeted harm. Participants emphasised that identifiers should be created with a secure and well-regulated system to prevent misuse of the hidden disability identification card.

#### Category 3: Participants’ attitudes toward the hidden disability advocacy campaign

Participants in the FGD were eager to engage in the discussion to advocate for hidden disability groups. Up until now, there have been no initiatives that allow hidden disability groups to voice their opinions. This activity is the first in Indonesia, designed to advocate for the rights and needs of individuals with hidden disabilities. Participants expressed enthusiasm and high hopes that the FGD would catalyse positive change, promoting greater inclusivity of public transportation for individuals with hidden disabilities.

### Summary

This study identified the following 3 themes from the FGD: (i) patients’ with ARD perceptions of their disease and treatment, (ii) experiences with public transportation accessibility in Jakarta, and (iii) views on the use of identifiers for individuals with hidden disabilities. Illustrative quotations supporting each theme are presented in Tables 2 to 4, and a summary of this study’s results is shown in Table 5. The primary themes were determined based on the frequency (theme percentage) of total codes.

**Table 5:** Summary of themes and categories

Theme	% Per total codes	Categories	% Per total codes	% Per theme
Subjects' perceptions of their disease and treatment	48%	Impact on daily life	22%	47%
		Unpleasant hospital visit experiences	13%	28%
		Coping attitudes	12%	25%
Subjects' perceptions of current public transportation accessibility	29%	Difficulties in using public transportation	14%	49%
		Public transportation infrastructure	12%	43%
		Public transportation personnel awareness	2%	7%
The use of identifiers for hidden disability groups	24%	Methods for identification	13%	54%
		Perceptions toward disability groups categorisation	9%	39%
		Attitudes toward the hidden disability advocacy campaign	2%	7%

The primary theme most frequently reflected participants’ concerns regarding their personal experiences with their disease and treatment. Within this theme, the impact on daily life emerged strongly, ranging from physical and emotional burdens to changes in daily routine. Other important aspects included unpleasant hospital visits and individual coping strategies. These emphasised the challenges of living with ARD as a hidden disability that affects not only physical function but also emotional well-being and social identity, reinforcing the need to include individuals with hidden disabilities in priority groups.

**Figure 2:**
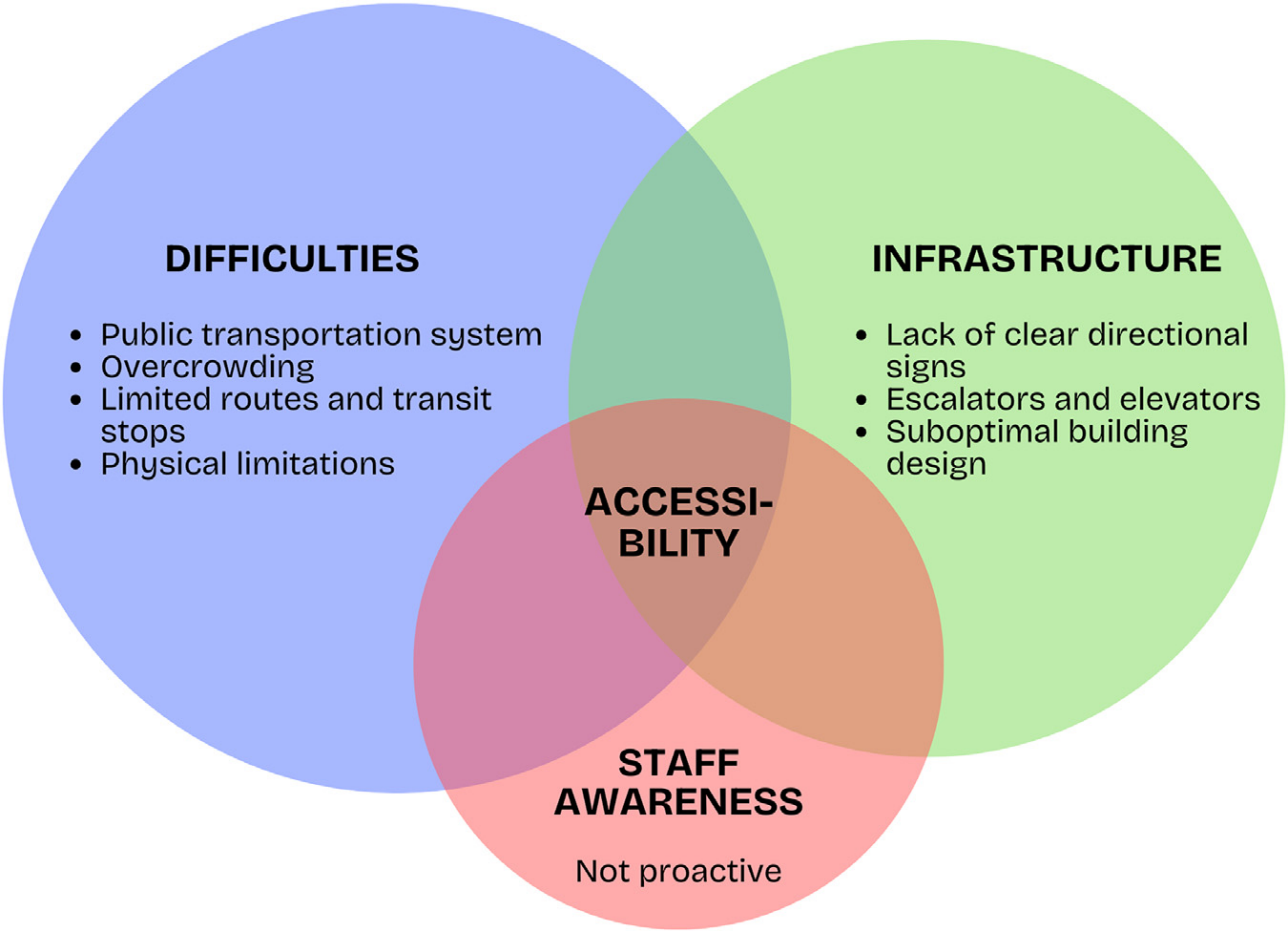
Illustration of elements affecting public transportation accessibility for patients with autoimmune rheumatic disease as a hidden disability group.

The second theme focused on participants’ experiences with public transportation, particularly the challenges in accessing facilities safely and efficiently. Respondents described difficulties in navigating overcrowded systems, inadequate infrastructure, and the inaccessibility of features such as escalators or signage. They also highlighted a lack of awareness among public transport personnel about the needs of passengers with invisible disabilities, suggesting a need for greater awareness of hidden disabilities. Elements affecting public transportation accessibility for patients with ARD, as a hidden disability group, are shown in Figure 2.

A third key theme explored the need for special identifiers to recognise individuals with hidden disabilities. Participants discussed both the potential benefits and risks of such identifiers, expressing concern about misuse and privacy while also advocating for improved recognition and support. Many viewed the focus group itself as an important first step in raising awareness and promoting advocacy for hidden disability inclusion in public infrastructure, such as public transportation.

## DISCUSSION

### Study participants' profile

This study involved 14 participants recruited through 8 ARD patient support groups, 4 of which were specific to SLE. SLE was the most common diagnosis among participants, reflecting its high prevalence and the greater availability of SLE support groups in Indonesia compared to those with other ARDs. Of the 14 participants, 13 were female and 1 was male, with ages ranging from 36 to 59 years, indicating they were in the productive age group. This demographic profile is consistent with national data on SLE in Indonesia, where the disease predominantly affects women and typically has its onset during the productive years of adulthood [9].

### Hidden disabilities in ARD

ARD encompasses chronic conditions that often result in visible physical changes [1]. For instance, SSc causes skin thickening and claw-like deformities, while ankylosing spondylitis leads to spinal deformities such as kyphosis [10,11]. These deformities can affect mobility, requiring walking aids or wheelchairs.

In addition to visible disabilities, patients with ARD frequently experience hidden disabilities, with most facing them throughout their lives. Symptoms such as chronic pain, fatigue, mobility issues, and cognitive dysfunction are often debilitating yet invisible to others, leading to ARD being classified as a ‘hidden disability.’

#### Slower cognitive processing and reduced pace in daily activities

Participants in this study reported needing to adjust their daily routines due to hidden disabilities, particularly slower cognitive processing and a reduced pace in activities. Cognitive impairment, commonly known as brain fog, affects 30% of patients with SjD and 25% of those with SLE [12,13]. Two participants described having to repeat instructions to understand basic tasks, with one attributing her reduced processing ability to SpA and SjD. The cognitive difficulties that she reported primarily affected attention and memory. No other participants in this study explicitly mentioned similar symptoms, suggesting that cognitive impairments may be underrecognised or not readily attributed to the disease by other patients. Although not always visible, cognitive difficulties limit patients’ with ARD participation and productivity in work, family, and social life [14,15]. Roughly 18.8% of individuals with rheumatic musculoskeletal diseases avoid disclosing their diagnosis at work due to stigma [16].

Lifestyle modifications further slow the pace of activities in patients with ARD. Regular exercise and nutritious diets are prioritised to prevent flares and manage disease severity [17]. To minimise fall risk, patients often slow their movements due to chronic pain, impaired postural balance, and restricted mobility [18].

#### Physical limitations

Progressive joint damage associated with ARD limits mobility, strength, and range of motion [19]. Many participants cited difficulty with commuting, especially in crowded spaces and navigating stairs. These findings are consistent with a study by Teuwen et al [20], which identified stair climbing, walking, posture changes, grasping, lifting, and standing as common limitations in patients with RA and SpA. One patient with SSc in this study also struggled with driving due to hand stiffness and Raynaud’s phenomenon, further impairing her hand function [21].

#### Emotional and psychological impact

Psychologically, participants reported heightened emotional sensitivity and loss of self-confidence, aligning with increased mental health risks in SLE, SjD, RA, and dermatomyositis [22]. Factors such as low socioeconomic status, inadequate coping mechanisms, and insufficient emotional support exacerbate these issues [23].

#### Treatment and coping mechanisms

Despite these challenges, participants demonstrated strong treatment adherence, aiming to reduce disease activity and maintain quality of life. Resilience—defined as adaptive coping—was associated with better outcomes [24].

However, hospital visits remain challenging. Long waiting times were a major concern, particularly for patients experiencing flare-ups, as fatigue exacerbates discomfort during these visits. The Indonesian national health insurance system, BPJS, covers up to 250 million Indonesians, equivalent to 90% of Indonesia’s entire population, leading to high patient loads and overcrowded hospitals. To reduce this burden, some patients with ARD use telemedicine for more convenient consultations and ongoing care [25].

In this study, only one participant mentioned using telemedicine as an option during periods when her disease was stable. This option allowed her to obtain regular medications conveniently without enduring long hospital queues. Globally, telemedicine has emerged as a growing method for delivering healthcare remotely. It has been shown to be cost-effective and, in many cases, comparable with traditional face-to-face care, despite certain limitations [26]. Although telemedicine could be a potential solution to reduce the need for patients to travel to hospitals, its use among patients with ARD in Indonesia remains uncommon. Currently, most commercial telemedicine platforms in Indonesia cater primarily to private insurance holders, and the Indonesian national health insurance system does not cover telemedicine services [25]. Hence, hospital visits continue to be the primary method through which patients with ARD access care.

### Challenges in commuting with public transportation for patients with ARD

Accessing public transportation in Jakarta is challenging for patients with ARD, especially those with hidden disabilities. A study revealed that 76% of newly diagnosed patients with RA encountered transportation challenges, primarily due to accessibility and cost, affecting care continuity [7]. Accessible transportation is essential for patients with ARD to attend medical appointments and manage daily activities. Mobility issues hinder boarding, stair climbing, and navigation through crowded or poorly designed environments.

Studies from various regions reinforce these concerns. In Nigeria, individuals with mobility impairments struggle with boarding, insufficient driver assistance, long wait times, and a lack of clear timetables and signage [27]. Similarly, Bezyak et al [28] highlighted inadequacies in public transport systems, including inaccessible stops and stations and insufficient driver training on disability needs. In Malaysia, people with disabilities in Kuala Lumpur reported negative perceptions of public transportation due to issues with procedures, vehicle design, and building layouts [29].

#### Individual responses to accessibility

Although most participants reported difficulties, individual responses varied. A SSc patient avoided riding a motorcycle due to numb fingertips and preferred the Commuter Line train for its comfort. A patient with SLE used public transport when feeling well but switched to ride-hailing services during flares, despite the cost. However, she emphasised that taxis were not always ideal, as sun exposure triggered burning sensations and traffic congestion led to mental exhaustion. These examples illustrate the diverse transportation needs among patients with ARD, shaped by fluctuating symptoms.

#### Lack of inclusivity in public transportation systems and infrastructure designs

Consistent with previous studies, the FGD revealed patients’ with ARD concerns about Jakarta’s public transportation, including overcrowding, limited routes and stops, inadequate disability-friendly facilities, and a lack of staff awareness about hidden disabilities. Supportive infrastructure, such as handrails, elevators, and escalators, could greatly assist those with mobility impairments, particularly patients with ARD experiencing joint pain. A study by A’Rachman et al [30] found that long walking distances between stops in Central Jakarta pose an additional barrier for people with disabilities.

Improving architectural accessibility is essential and should include features such as high-contrast signage with large fonts, clear wayfinding, adequate lighting, and safe platform designs [31]. Hazards such as platform gaps and slippery surfaces can be minimised by following established standardised guidelines, such as the Americans with Disabilities Act standards [32].

#### Lack of general public and transport personnel awareness

The unpredictable nature of ARD means patients may appear well during remission but experience disabling flare-ups at other times. The invisibility of these symptoms often limits public understanding and support. Without visible indicators, patients with ARD frequently face scepticism or microaggressions when requesting accommodations such as priority seating [33].

Studies have highlighted the limited awareness among transport personnel and passengers, forcing individuals with hidden disabilities to subtly signal their need for support [34]. Conflicts may also arise when they must compete with other priority groups for limited accessible spaces [35]. To create a more inclusive environment, comprehensive training is needed for transport staff to recognise and support passengers with hidden disabilities [31].

Indonesia has issued disability-related regulations, including Act No. 8/2016 and Government Regulation No. 17/2019. Specific provisions for transport accessibility appear in Government Regulation No. 42/2020 and Ministry of Transportation Regulation No. PM 98/2017. However, these policies primarily address visible disabilities. People with hidden conditions such as ARD are not yet explicitly included among those granted accessibility rights.

### Special identifiers for patients with ARD as a hidden disability group

To grant priority rights to individuals with hidden disabilities, an identification system is necessary to recognise this group. Lanyards, pins, or badges have been used globally to indicate specific needs discreetly, especially in transport settings. Such identifiers help bridge communication gaps and could be supported by clear signage near priority seating areas [34].

In Indonesia, special identifiers are currently limited to specific priority groups, such as pregnant women [36]. In contrast, other countries have adopted identifiers for various hidden disabilities, ranging from autism to chronic illnesses, such as the Hidden Disabilities Sunflower Lanyard in the UK, USA, and others [37], the Verified Hidden Disability Lanyard (UK), and the Invisible Disabilities Australia Access Card [38,39].

Findings from this study highlight the need for a secure, government-regulated identifier system, ideally integrated with the Ministry of Health database to prevent misuse. Most existing international systems are nongovernment organisation-led and not integrated with official health registries [[37], [38], [39]], making them susceptible to unregulated use despite their benefits in improving visibility.

Some respondents in this study expressed their concerns about using visible identifiers, such as badges or symbols, as it would publicly identify them as disabled. These concerns were primarily related to stigma, privacy, and the potential for unwanted attention. This highlights a tension between needing support and fearing stigma. Although participants acknowledged the potential benefits of such identifiers in specific situations—such as gaining access to priority seating on public transportation or receiving assistance from staff—they also emphasised the importance of maintaining the confidentiality of their personal health information and diagnosis.

This study has several limitations. First, it primarily focuses on patients with ARD and does not include perspectives from transport operators, policymakers, caregivers, or other commuters. Second, the study focuses exclusively on the Jakarta Metropolitan Area, which has the most developed public transportation system in Indonesia, and may not reflect conditions in other regions. Third, we did not analyse accessibility for hidden disability groups in other cities. However, as we included ARD support groups with members from across Indonesia, we hope that the findings of this study can contribute to advocacy efforts in other regions as well. Future research could explore a more comprehensive analysis of the needs of individuals with hidden disabilities, especially patients with ARD, in other public spaces such as hospitals, public buildings, and workplaces.

## CONCLUSION

Although Jakarta has notably progressed in enhancing public transport accessibility for individuals with disabilities, significant gaps persist, particularly for those with hidden disabilities such as patients with ARD. Hidden disabilities can profoundly affect various aspects of daily life, including the ability to commute using public transportation. Patients with ARD often encounter challenges due to an underdeveloped public transportation system, leading to limited routes and overcrowding, which make commuting difficult for those with hidden disabilities. Additionally, poorly designed infrastructure fails to accommodate their mobility needs, and transport personnel often lack adequate training to recognise hidden disabilities as a priority group requiring assistance. This study emphasises the importance of introducing special identifiers to help signal the needs of individuals with hidden disabilities to both the general public and transport personnel. The adoption of such identifiers could enhance awareness and promote more inclusive practices in public transportation.

To bridge the accessibility gaps, comprehensive assessments and the implementation of inclusive policies are crucial. Collaborative efforts involving government agencies, urban planners, healthcare providers, and key stakeholders are essential to create a public transport system that genuinely accommodates the needs of all community members, including those with hidden disabilities.

## Competing interests

The authors declare no conflict of interest.

## Appendix Supplementary materials

Image, application 1

Image, application 2

Image, application 3
