# Supplementary material for: Hidden disabilities in patients with autoimmune rheumatic disease: an invisible barrier to daily functioning and healthcare accessibility
Source: EULAR Rheumatol Open. 2025 Sep 6;1(3):237–48. doi: 10.1016/j.ero.2025.08.003 (PMC13292421; doi:10.1016/j.ero.2025.08.003)
Supplement: Supplementary file 1 [file mmc1.pdf]

Manuscript Title: Hidden Disabilities in Autoimmune Rheumatic Diseases Patients: an Invisible Barrier to Daily Functioning and Healthcare Accessibility
Corresponding Author: Faisal Parlindungan

Signatures of all authors

Name of Author	Date	Signature
Faisal Parlindungan	March 16th, 2025	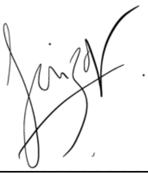
Rudy Hidayat	March 16th, 2025	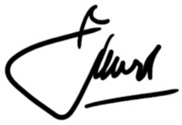
Sumariyono	March 16th, 2025	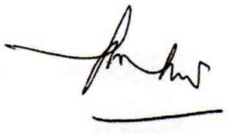
Suryo Anggoro Kusumo Wibowo	March 16th, 2025	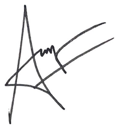
Anna Ariane	March 16th, 2025	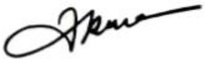
Johanda Damanik	March 16th, 2025	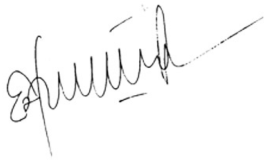
Abirianty Priandani Araminta	March 16th, 2025	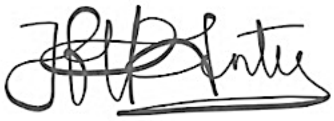
Cindy	March 16th, 2025	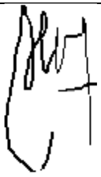

Mitra Alparisa	March 16th, 2025	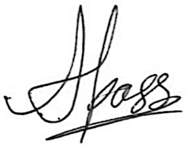
Tamariska Evelyn	March 16th, 2025	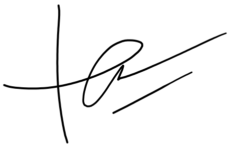
Indra Saputra	March 16th, 2025	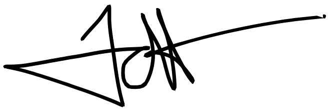
Annisa Aulia Fitri	March 16th, 2025	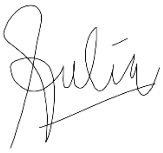
Rio Rialdi	March 16th, 2025	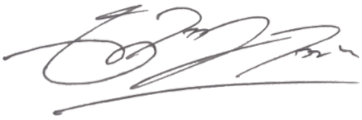

Authors' Contributions

Conceptualisation	1. Faisal Parlindungan<br>2. Rudy Hidayat<br>3. Suryo Anggoro Kusumo Wibowo
Data curation	1. Faisal Parlindungan<br>2. Mitra Alparisa<br>3. Tamariska Evelyn
Formal analysis	1. Faisal Parlindungan<br>2. Mitra Alparisa<br>3. Tamariska Evelyn
Investigation	1. Faisal Parlindungan<br>2. Tamariska Evelyn<br>3. Mitra Alparisa<br>4. Johanda Damanik<br>5. Abirianty Priandani Araminta
Methodology	1. Faisal Parlindungan<br>2. Cindy Oey<br>3. Tamariska Evelyn
Project administration	1. Faisal Parlindungan<br>2. Annisa Aulia Fitri<br>3. Rio Rialdi<br>4. Indra Saputra
Resources	1. Faisal Parlindungan<br>2. Cindy Oey

	3. Annisa Aulia Fitri<br>4. Rio Rialdi<br>5. Indra Saputra
Software	1. Faisal Parlindungan<br>2. Cindy Oey<br>3. Mitra Alparisa<br>4. Tamariska Evelyn
Visualisation	1. Mitra Alparisa<br>2. Tamariska Evelyn
Writing – original draft	1. Faisal Parlindungan<br>2. Mitra Alparisa<br>3. Tamariska Evelyn<br>4. Anna Ariane
Writing – review and editing	1. Faisal Parlindungan<br>2. Rudy Hidayat<br>3. Sumariyono<br>4. Suryo Anggoro Kusumo Wibowo
