# Supplementary material for: Hidden disabilities in patients with autoimmune rheumatic disease: an invisible barrier to daily functioning and healthcare accessibility
Source: EULAR Rheumatol Open. 2025 Sep 6;1(3):237–48. doi: 10.1016/j.ero.2025.08.003 (PMC13292421; doi:10.1016/j.ero.2025.08.003)
Supplement: Supplementary file 3 [file mmc3.docx]

**Focus Group Guide**

| **PREPARATION** | | |
| --- | --- | --- |
| 1. | Identify and confirm participants, and establish a mutually agreed schedule. |  |
| 2. | Prepare the FGD team, including trained facilitators/moderators and a designated recorder. |  |
| 3. | Arrange the FGD venue and ensure necessary equipment is available, such as an audio recorder, writing materials, and appropriate room setup. |  |
| 4. | Check transportation arrangements for the research team to the venue. |  |
| 5. | Provide meals or refreshments if necessary. |  |
| 6. | Prepare and test the recording device, including a backup recorder. Check batteries and bring chargers and extension cords. |  |
| 7. | Prepare notebooks and pens for note-taking. |  |
| 8. | Organize all documents and materials in one container (e.g., a bag or folder), including:   - FGD guide sheet - Informed consent forms (according to the number of participants) - Information sheets for participants - Participant incentives and corresponding receipts |  |
| 9. | Revisit the research topic and FGD guide. Anticipate possible participant questions and prepare appropriate, diplomatic responses. Discuss potential participant concerns with the research team during a brainstorming session. |  |
| 10. | Review the content of the participant information sheet to ensure clarity and consistency. |  |
| 11. | Conduct a rehearsal session or dry run of the FGD. |  |
| 12. | Demonstrate your commitment to the participants by arriving on time as scheduled, and ensuring all materials (recorders and backups, interview guide, consent forms, notebook, and working pens with backups) are ready and available. |  |
| **IMPLEMENTATION** | | |
| 13. | Greet participants and engage in light, informal conversation to help create a comfortable atmosphere. Use appropriate humor when necessary. |  |
| 14. | Introduce the facilitator and note-taker/recorder to the participants. |  |
| 15. | Clearly explain the purpose and process of the FGD. |  |
|  | Present and obtain informed consent before beginning the session. This should include:   - An explanation of the study and its objectives - The role of participants - The use of audio (and/or visual) recordings and written notes - Confidentiality assurance - Potential risks and benefits - Expected duration of the session - The right of participants to pause or withdraw at any time during the discussion - An opportunity for participants to ask questions |  |
| 16. | Begin audio recording once consent has been obtained. |  |
| 17. | Facilitate the discussion in a relaxed and engaging manner. Use appropriate humor when necessary to maintain a comfortable and open environment. Emphasize that there are no right or wrong answers. |  |
| 18. | For sensitive questions, participants may hesitate to respond. If the discussion stalls, gently prompt by referring to opinions or findings from previous FGDs or relevant sources. Offer the option to write down their responses if they are uncomfortable speaking. |  |
| 19. | Take notes on areas that require further probing. Apply both verbal and non-verbal probing techniques, such as:   - Repeating or paraphrasing participants’ responses - Asking follow-up or clarifying questions - Using silence, eye contact, and nodding to encourage deeper reflection |  |
| 20. | Ask the note-taker if there are any points that need further exploration. |  |
| 21. | Conclude the FGD by summarizing the key points discussed and thanking participants for their contributions. |  |
| 22. | Turn off the audio recorder. |  |
| **DOCUMENTATION** | | |
| 23. | Record the FGD session using more than one device (audio, video, or photographs) to ensure data backup. |  |
| 24. | Take manual notes during the session as an additional backup. |  |
| 25. | Observe the session actively and document relevant contextual information (e.g., group dynamics, setting, interruptions). |  |
| 26. | Immediately after the FGD, expand and complete the field notes while the memory of the session is still fresh. |  |
| **RESPONSIBILITIES OF THE RECORDER/NOTE-TAKER** | | |
| 27. | The designated note-taker is responsible for recording the following:   - Date, time, and location of the session - General dynamics and atmosphere of the discussion - Participant characteristics (e.g., group interaction, expressions of engagement or hesitation) - Noteworthy or unique opinions raised during the session - Emotional tone and expressions of participants - Language use, including informal or culturally specific terms - Spontaneous comments made during breaks or after the formal session concludes |  |
| **THINGS TO BRING** | | |
| 28. | - Notebook for manual note-taking and session documentation - Copies of the informed consent forms - Audio/video recordings and clearly labeled backup notes - Incentive receipts and relevant documentation for participant compensation - All other tools or materials used during the FGD session (e.g., pens, printed guides, cue cards) |  |

**Pilot Questions for FGD**

| **NO** | **PROMPTS** | **CHECKLIST** |
| --- | --- | --- |
|  | General question: Can you describe the illness you are currently experiencing? |  |
| 1. | In your opinion, how does your illness limit your daily activities? How do you think your illness affects your day-to-day functioning? |  |
| 2. | How do you feel about the obligation to regularly visit the hospital for treatment? |  |
| 3. | In your opinion, is the journey to the hospital comfortable? What has your experience been like when traveling to the hospital for check-ups? |  |
| 4. | How do you feel when using public transportation? |  |
| 5. | Do you think the current design of public transport buildings, stations, or shelters accommodates your needs? What is your perception of the current design of public transport buildings, stations, or shelters? |  |
| 6. | Do you think public transportation staffs are accommodating and helpful in assisting your access to public transportation? |  |
| 7. | Do you think hospital facilities are sufficient to support accessibility during treatment? What is your opinion on hospital facilities regarding your mobility during treatment? |  |
| 8. | Do you think hospital staff provide adequate support for accessibility during treatment? What is your opinion on hospital staff in relation to your mobility during treatment? |  |
| 9. | Do you think using a hidden disability identifier is necessary to help improve accessibility? What is your opinion on the use of a hidden disability identifier to support accessibility? |  |
| 10. | In your opinion, what is the best way to implement disability identifiers in public transportation? |  |

**FGD Topics for Moderators**

| **NO** | **PROMPTS** | **CHECKLIST** |
| --- | --- | --- |
| 1. | How do you think your illness limits your daily activities? |  |
| 2. | How do you feel about the obligation to regularly visit the hospital for treatment? |  |
| 3. | In your opinion, is the journey to the hospital comfortable enough? |  |
| 4. | How do you feel when using public transportation? |  |
| 5. | Do you think the current design of public transport buildings/stations/shelters accommodates your needs? |  |
| 6. | Do you think public transportation staffs are accommodating and helpful in assisting your access to public transportation? |  |
| 7. | In your opinion, is it necessary to use a hidden disability identifier to help with accessibility? |  |
| 8. | What do you think is the best way to use a hidden disability identifier in public transportation? |  |
